# Developmental disorders caused by haploinsufficiency of transcriptional regulators: a perspective based on cell fate determination

**DOI:** 10.1242/bio.058896

**Published:** 2022-01-28

**Authors:** Roman Zug

**Affiliations:** Department of Biology, Lund University, 22362 Lund, Sweden

**Keywords:** Haploinsufficiency, Developmental disorder, Transcription factor, Chromatin regulator, Positive feedback, Cooperativity

## Abstract

Many human birth defects and neurodevelopmental disorders are caused by loss-of-function mutations in a single copy of transcription factor (TF) and chromatin regulator genes. Although this dosage sensitivity has long been known, how and why haploinsufficiency (HI) of transcriptional regulators leads to developmental disorders (DDs) is unclear. Here I propose the hypothesis that such DDs result from defects in cell fate determination that are based on disrupted bistability in the underlying gene regulatory network (GRN). Bistability, a crucial systems biology concept to model binary choices such as cell fate decisions, requires both positive feedback and ultrasensitivity, the latter often achieved through TF cooperativity. The hypothesis explains why dosage sensitivity of transcriptional regulators is an inherent property of fate decisions, and why disruption of either positive feedback or cooperativity in the underlying GRN is sufficient to cause disease. I present empirical and theoretical evidence in support of this hypothesis and discuss several issues for which it increases our understanding of disease, such as incomplete penetrance. The proposed framework provides a mechanistic, systems-level explanation of HI of transcriptional regulators, thus unifying existing theories, and offers new insights into outstanding issues of human disease.

This article has an associated Future Leader to Watch interview with the author of the paper.

## Abstract (precis)

**Summary:** This Review proposes the hypothesis that developmental disorders caused by haploinsufficiency of transcriptional regulators result from disrupted bistability (i.e. disrupted positive feedback or cooperativity) in the gene-regulatory network of the underlying cell fate decision.

## Introduction

During human development, a single cell gives rise to hundreds of different cell types that become functionally organized into distinct organs and tissues. Such an astonishing instance of morphogenesis requires recurrent specification of different cell fates to ultimately establish tissue and organ identities. Hence, disruptions of cell differentiation and fate specification can lead to a broad range of developmental disorders (DDs) (see Glossary, Box 1), which most evidently manifest as congenital malformations and neurodevelopmental disorders (NDDs) (for both terms, see Glossary, Box 1). DDs are a significant burden to human health. Congenital malformations are the number one cause of infant mortality in the US (Wallingford, 2019), and NDDs such as intellectual disability (ID) and autism spectrum disorder (ASD) are among the leading socio-economic health care problems in Western countries (Ropers, 2010; Lyall et al., 2017). In order to develop better treatments, it is crucial to gain a mechanistic understanding of these disorders.

**Box 1. Glossary**

**Biomolecular condensates:** intracellular membraneless compartments that concentrate proteins and/or nucleic acids and that are formed by liquid–liquid phase separation. Super-enhancer (SE)-associated condensates consisting of TFs, cofactors and RNA polymerase II are called transcriptional condensates.

**Bistability:** having two stable steady states, and one unstable one (a threshold), for a single input value.

**Cis-regulatory element (CRE):** a noncoding DNA region that regulates gene transcription, typically by binding to TFs.

**Cofactors:** regulatory proteins that typically do not bind to DNA directly but are required for proper gene expression by mediating interactions between TFs, other cofactors, and the basal transcriptional machinery. Frequently, cofactors function as chromatin regulators – large protein complexes with enzymatic activity –, which enables them to impact gene regulation through chromatin remodelling. Hence, in this article, I use the terms ‘cofactor’ and ‘chromatin regulator/remodeler’ more or less synonymously.

**Congenital malformation (birth defect):** a developmental disorder (DD) that results from cell fate errors during embryogenesis. Congenital malformations are physical defects recognizable at birth and can affect basically any organ system.

**Cooperativity:** a fundamental principle governing the binding of master TFs to DNA. Under cooperative binding, one binding event facilitates a subsequent one; in turn, multiple binding events have a greater-than-additive (synergistic) effect on transcriptional output, leading to a sigmoidal (switch-like) transcriptional response.

**Developmental disorder (DD):** a disorder arising from defects in the development of one or several organ systems (in the latter case, the DD is often called a syndrome). Frequently, DDs are caused by genetic variants that disrupt cell differentiation and fate determination. Within the scope of this article, DDs include congenital malformations, NDDs, and disorders of maintenance and self-renewal.

**Disorder of maintenance and self-renewal:** a DD that affects tissue homeostasis, maturation, or regeneration after birth.

**Enhancer**: a CRE that can be bound by TFs to enhance transcription.

**Gene regulatory network (GRN):** the interactions between TF genes and their CREs to process input signals into cellular output functions.

**Haploinsufficiency (HI):** the production of a dominant phenotype in diploid organisms that are heterozygous for a loss-of-function allele; in other words, a 50% level of function is not sufficient for a normal phenotype.

**Incomplete penetrance:** in medical genetics, the phenomenon that not all carriers of a disease-causing mutation actually develop the disease.

**Intrinsically disordered proteins (IDPs)** and **intrinsically disordered regions (IDRs):** proteins and protein regions that contain extensive disordered (unstructured) sequences that are important for function. These sequences are often characterized by low complexity, exhibiting lower amino acid diversity than most protein sequences, for example due to short sequence repeats.

**Neurodevelopmental disorder (NDD):** a DD that affects the development of the nervous system, leading to abnormal brain function.

**Nonsense-mediated mRNA decay (NMD):** a cellular quality control pathway that degrades mRNAs that contain premature termination codons as a result of nonsense or frameshift mutations.

**Phase separation:** the demixing of a homogenous mixture into two coexisting phases. In the case of liquid–liquid phase separation, a homogenous solution separates into two liquid phases.

**Positive feedback:** in gene regulation, a process in which a gene activates or maintains its own expression, either directly or indirectly, including double-negative feedback.

**Positive feedback with cooperativity (PFC):** a key GRN motif for cell fate determination, consisting of master TFs that activate their own expression by cooperatively binding to their own SEs.

**Super-enhancer (SE):** a cell type-specific enhancer cluster that contains large numbers of TF binding sites and that concentrates high densities of master TFs, cofactors and components of the basal transcriptional machinery, including RNA polymerase II.

**Transcription factors (TFs):** regulatory proteins that bind to specific DNA sequences within enhancers (TF binding sites) through their DNA-binding domains (DBDs) and that activate or repress transcription by recruiting and interacting with other TFs and cofactors through their activation domains. TFs in control of cell fate are also called master TFs or lineage-determining TFs.

**Ultrasensitivity:** a property of input–output relationships that makes them switch-like in character.

**Variable expressivity:** in medical genetics, the phenomenon that individuals with a disease phenotype often differ in the degree to which the phenotype is expressed.

Cell fate determination requires appropriate spatial and temporal expression of specific genes. Consequently, many DDs arise from impaired gene regulation, a frequent cause being loss-of-function mutations in genes coding for transcription factors (TFs) and cofactors (for both terms, see Glossary, Box 1), as well as in the cis-regulatory elements (CREs) (see Glossary, Box 1) of these genes (Lee and Young, 2013; Fahrner and Bjornsson, 2019; Van der Lee et al., 2020). Strikingly, however, these genes commonly exhibit a distinct dosage sensitivity, such that heterozygous loss-of-function mutations are sufficient to elicit a DD. This dosage sensitivity is called haploinsufficiency (HI) (see Glossary, Box 1; Box 2). HI represents a form of dominance, as the loss-of-function mutation in the variant allele is dominant to the wild-type allele. This stands in stark contrast to the majority of disease-causing mutations, which are recessive (Jimenez-Sanchez et al., 2001; Kondrashov and Koonin, 2004). Recent large-scale sequencing efforts to identify human genes that are intolerant to or under strong selection against heterozygous loss-of-function are sparking tremendous interest in HI and its role in disease etiology (Lek et al., 2016; Kaplanis et al., 2020; Karczewski et al., 2020).

**Box 2. Genetics of HI**

HI can arise from reduced gene dosage, expression, or protein activity (Wilkie, 1994), and through both variation in coding and non-coding regions of DNA (Box 2-Figure):

1. Structural variation, both unbalanced and balanced, can decrease gene dosage (Weischenfeldt et al., 2013; Spielmann et al., 2018). For example, gene deletions are loss-of-function by definition. But also balanced rearrangements such as translocations can disrupt coding sequences and thus lead to reduced gene dosage (Box 2-Figure B).
2. Nonsense (stop-gain) and frameshift mutations (which together are often subsumed as protein-truncating variants) introduce premature stop codons, which usually results in the degradation of mRNA by nonsense-mediated decay (NMD) (see Glossary, Box 1) (Lykke-Andersen and Jensen, 2015), leading to reduced gene expression (Box 2-Figure C).
3. Missense mutations, splice site mutations, and small in-frame indels that inactivate functional protein domains (rather than exert a dominant negative effect or lead to gain-of-function) will lead to reduced protein activity (Box 2-Figure D). In addition, transcripts involving premature stop codons sometimes escape NMD so that truncated proteins will be produced, which may also have reduced activity if a functional domain is affected.
4. Finally, structural variation and single-nucleotide variants can also lead to reduced gene expression via deletion, disruption, or disconnection of CREs (Box 2-Figure E). Such regulatory loss of function, including the phenomenon of position effect, is a well-known cause of DDs (Chatterjee and Ahituv, 2017; Spielmann et al., 2018; Laugsch et al., 2019).

**Box 2-Figure.**
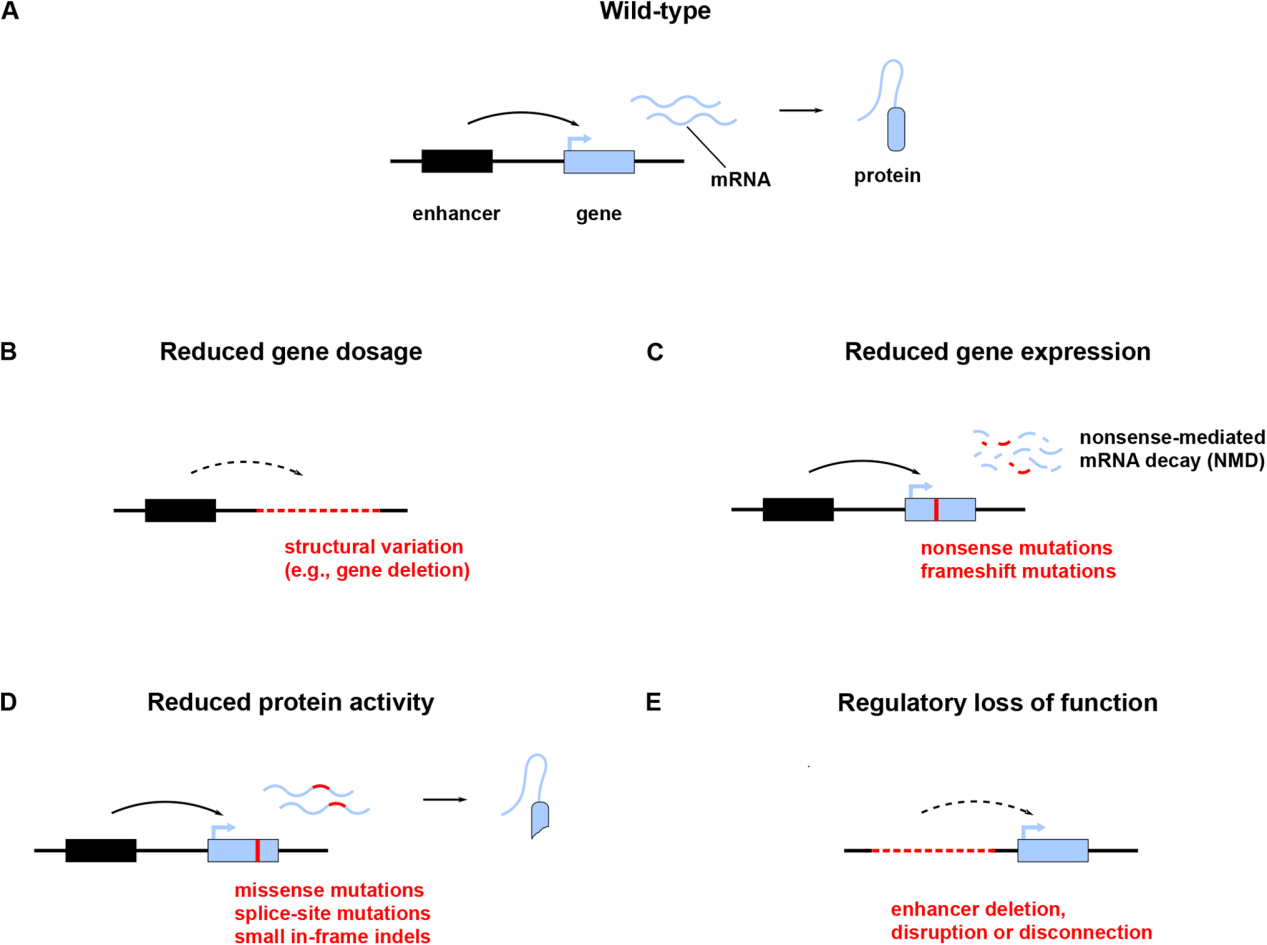
(A) Wild-type gene expression leads to the production of a functional protein (only one allele is shown throughout the figure). (B) Structural variation such as gene deletions leads to reduced gene dosage. (C) Nonsense and frameshift mutations produce premature stop codons, which results in nonsense-mediated mRNA decay (NMD), leading to reduced gene expression. (D) Missense mutations, splice-site mutations, and small in-frame indels that inactivate protein domains, rather than exert a dominant negative effect, lead to reduced protein activity. (E) Deletion, disruption, or disconnection of enhancers also leads to reduced gene expression (regulatory loss of function).

Why are loss-of-function mutations in a single gene copy sufficient to cause DDs? And what makes TF/cofactor genes particularly prone to HI? In this article, I attempt to provide a unified explanation of this phenomenon. I argue that the regulatory architecture of cell fate determination makes TF/cofactor dosage sensitivity an inherent property of fate decisions. In particular, I propose the hypothesis that DDs caused by TF/cofactor HI result from disruption of bistability (see Glossary, Box 1) in the gene regulatory network (GRN) (see Glossary, Box 1) underlying the fate decision. Since bistability requires both positive feedback and cooperativity (or, more generally, ultrasensitivity; for all three terms, see Glossary, Box 1) (Ferrell, 2002), disrupting either is sufficient to cause disease. I present evidence in support of the proposed framework, including a simple theoretical model, and discuss important insights and implications for puzzling phenomena such as incomplete penetrance and variable expressivity (for both terms, see Glossary, Box 1) and for the etiology of complex disorders.

## Hundreds of DDs are caused by HI of transcriptional regulators

The recognition of DDs as ‘inborn errors of morphogenesis’ and the search for their genetic causes dates back at least half a century (Smith, 1966; Holmes, 1974). Since the 1980s, chromosomal microdeletions have been associated with DDs, for example the 22q11 deletion with DiGeorge syndrome, and the 17p11 deletion with Smith­­–Magenis syndrome (Watson et al., 2014). In the 1990s, it became evident that DDs are often caused by HI of TF genes, such as aniridia caused by *PAX6* HI (Ton et al., 1991) or Waardenburg syndrome caused by *PAX3* HI (Tassabehji et al., 1992; Baldwin et al., 1992). In fact, for many microdeletion syndromes, HI of a specific transcriptional regulator gene could later be pinpointed as the causal factor for most of the characteristic features, for example *TBX1* HI in DiGeorge syndrome (Yagi et al., 2003) and *RAI1* HI in Smith–Magenis syndrome (Slager et al., 2003). To date, HI of transcriptional regulator genes or their CREs has been determined as the underlying cause of a plethora of DDs (Fisher and Scambler, 1994; Engelkamp and van Heyningen, 1996; Seidman and Seidman, 2002; Angelozzi and Lefebvre, 2019; Fahrner and Bjornsson, 2019; Van der Lee et al., 2020; Cenik and Shilatifard, 2021; Herman et al., 2021). A comprehensive screening of the literature revealed more than 200 DDs that are caused by HI of TFs and cofactors that function as master regulators of the organ system(s) or cell lineage(s) affected in the respective disorder (see Tables S1–S3).

An overview of DDs caused by TF/cofactor HI is given in Table 1, which groups them into three categories, depending on when and where cell fate specification goes wrong (and acknowledging that many syndromes defy clear categorization). The first category consists of congenital malformations; common examples include congenital heart defects (CHDs) and limb malformations. But development does not stop at birth. Given the long period of postnatal brain development (Silbereis et al., 2016), NDDs may appear only later in life and therefore constitute a second category; common examples include ID and ASD. Finally, cell fate determination also plays a vital role in developmental processes such as tissue homeostasis, maturation, and regeneration (for such a broad conception of development, see, for example, Bilder, 2016). Cell fate errors that occur during these processes give rise to what I term disorders of maintenance and self-renewal (see Glossary, Box 1). Such disorders constitute the third category; examples include cancer predisposition syndromes and immunodeficiencies.

**Table 1.**
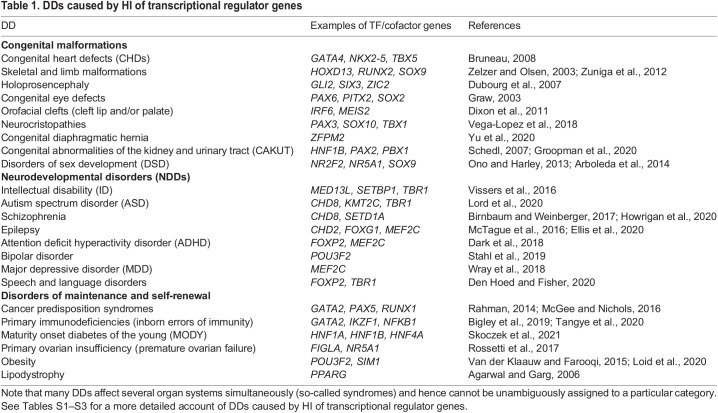
DDs caused by HI of transcriptional regulator genes

It is important to note that most of the disorders listed in Table 1 can also be caused by environmental factors, or by mutations in genes that are not involved in cell fate determination. Nevertheless, for each disorder listed, there are transcriptional regulator genes involved in fate specification for which variants of large effect size have been associated with the disorder. Some examples of these genes are also given in Table 1. What these genes have in common is their HI: heterozygous loss-of-function is sufficient to cause disease.

## Current thinking about the causes of HI

Existing theories to explain the mechanisms of HI can be broadly grouped into two categories: the insufficient amounts hypothesis and the dosage balance hypothesis (Johnson et al., 2019; Morrill and Amon, 2019). While the former states that the reduced amount of protein being produced is insufficient to perform its normal function, the latter attributes HI to stoichiometric imbalance of members in a protein complex. Here I briefly discuss both hypotheses, highlighting what each is contributing to a systems-level explanation of HI of transcriptional regulators involved in cell fate determination, but also what is missing. Such an explanation, I will argue in the next section, is provided by the concept of bistability, a crucial feature of biological systems involved in all-or-none processes such as cell fate decisions.

The insufficient amounts hypothesis posits that transcriptional regulators work close to a threshold level, thus rendering the system extremely dosage sensitive (Wilkie, 1994; Veitia, 2002; Johnson et al., 2019). In order to explain the threshold effect, the hypothesis makes reference to TF cooperativity (Veitia, 2002; Johnson et al., 2019) and positive feedback (Ferrer, 2002; Johnson et al., 2019), but treats each property separately, not recognizing that both are necessary to render a system bistable. Notably, Ferrer (2002) invokes bistability to explain HI of *HNF1A* and *HNF4A* leading to maturity-onset diabetes of the young (MODY). However, he focuses only on positive feedback and ignores cooperativity, failing to account for both requirements of bistability.

The dosage balance hypothesis focuses on protein complexes of defined stoichiometry; HI is explained as a consequence of subunit imbalance (Wilkie, 1994; Veitia, 2002; Johnson et al., 2019). In the context of transcriptional regulation, this hypothesis applies to TF dimers, and, in principle, to any factors that assemble cooperatively into a heteromeric complex of defined stoichiometry. However, cooperativity alone (that is, without positive feedback) is insufficient to produce bistability.

Taken together, both hypotheses include essential parts of a systems-level understanding of cell fate determination, in particular positive feedback and cooperativity, and thus lay the groundwork for understanding HI of transcriptional regulators. However, existing hypotheses do not bring positive feedback and cooperativity together, failing to recognize that both are necessary for proper cell fate determination, and hence cannot fully explain why disrupting either is sufficient to cause disease. In the next section, I attempt to provide such a unified explanation.

## A unified explanatory framework

Here, I bring together insights of previous theories to present a unified explanation of the HI of transcriptional regulators. This explanation is based on a systems-biology perspective, which regards cell fate decisions as bistable switches, requiring both positive feedback and cooperativity. Hence, I propose that HI of transcriptional regulators, as well as associated DDs, result from disruption of bistability (i.e. disruption of positive feedback or cooperativity) in the GRN underlying the cell fate decision.

### Cell fate decisions as bistable switches

Recent single-cell analyses have revealed with unprecedented resolution the details of cell fate determination. In short, cell fate choices are the outcome of quantitative changes in the concentrations of competing master TFs, gradually shifting cells toward fate commitment. Importantly, notwithstanding this gradual shift toward commitment, cell fate choices are inherently binary decisions. This has been shown, for example, for hematopoiesis (Palii et al., 2019), neurogenesis (Faure et al., 2020), neural crest development (Soldatov et al., 2019), pancreas development (Yu et al., 2019), and gonad development (Stévant et al., 2019). From a systems biology perspective, binary decisions are made by bistable systems; such a system can toggle between two alternative stable states but cannot rest in intermediate states (Ferrell, 2002). The concept of bistability in biological systems originates from pioneering work on gene regulation (Delbrück, 1949; Monod and Jacob, 1961; Kauffman, 1969). Since then, bistable switches have been identified as a paradigmatic GRN motif directing cell fate (Enver et al., 2009; MacArthur et al., 2009; Zhou and Huang, 2011; Ferrell, 2012).

Bistability requires two basic ingredients (Ferrell, 2002). The first is some form of positive feedback, which prevents the system from resting in intermediate states. The second is a mechanism to filter small stimuli out of the feedback loop, allowing the system to have a stable off state. This is usually achieved by some type of nonlinearity within the feedback loop, that is, some members of the feedback loop must behave in an ‘ultrasensitive’ manner. Ultrasensitivity means that, within a narrow range of inputs, the system will be extremely sensitive to input change, leading to a steep sigmoidal dose-response curve. A common source of ultrasensitivity is cooperativity. But how do both requirements for bistability, positive feedback and cooperativity, materialize in the process of cell fate determination?

### The significance of positive feedback and cooperativity in cell fate decisions

**Fig. 1.**
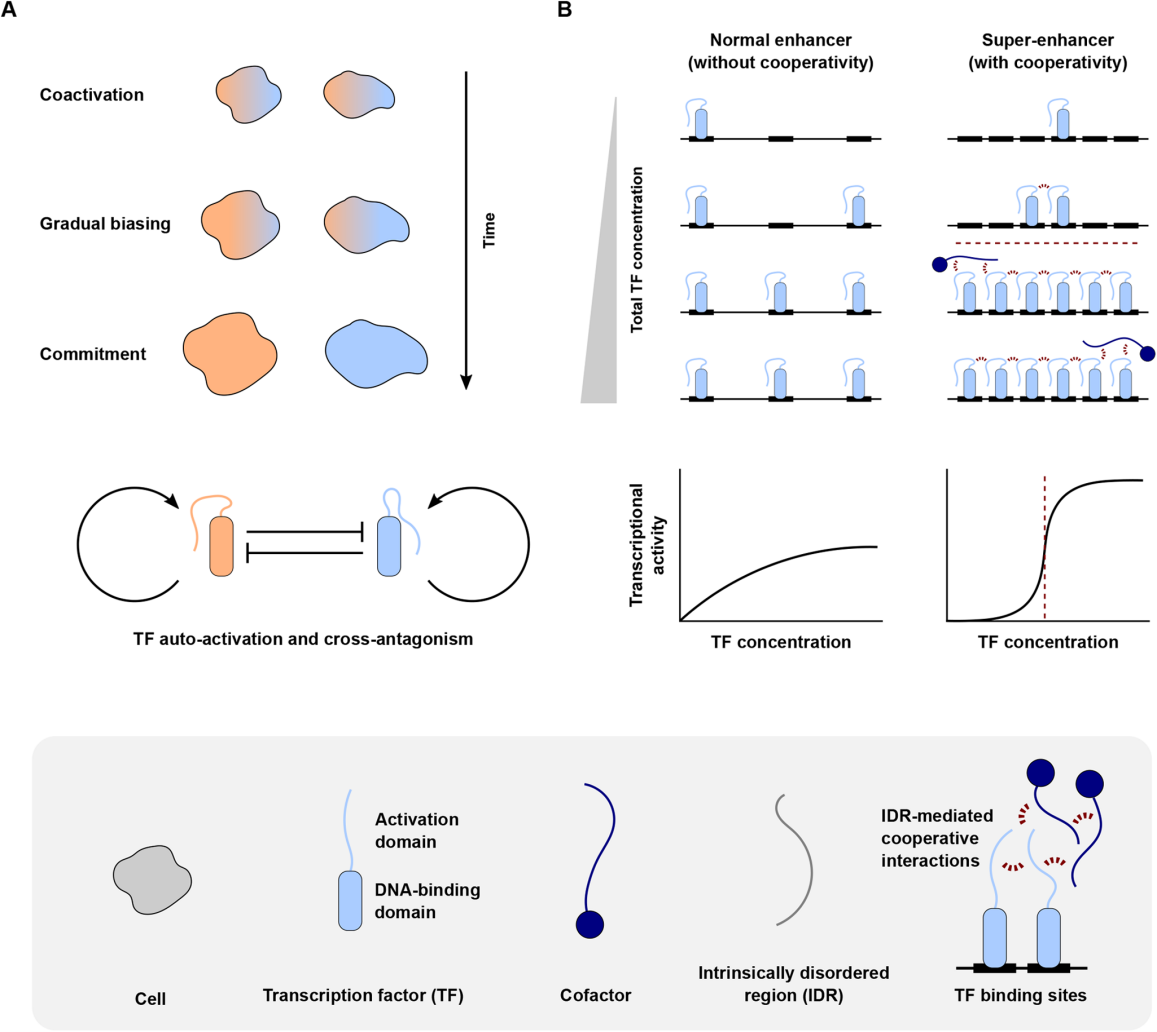
**The significance of positive feedback and cooperativity for cell fate decisions.** (A) The three phases of cell fate determination. Initially, master TFs from competing lineages are co-expressed in progenitor cells (coactivation phase). TF levels then change gradually in favor of one of the competing cell fate programs (gradual biasing phase). Finally, mutually exclusive gene expression programs commit each cell's fate (commitment phase). The process of fate determination is driven by master TFs both reinforcing their own expression program (auto-activation) and repressing competing fate programs (cross-antagonism). (B) At normal enhancers with a limited number of TF binding sites, TFs usually bind in a noncooperative manner, resulting in a gradually increasing transcriptional output. Conversely, super-enhancers with a large number of TF binding sites allow for cooperative TF binding, generating a sigmoidal (switch-like) transcriptional response. Part B adapted from Giorgetti et al. (2010).

The temporal dynamics of cell fate determination can be divided into three phases: coactivation, gradual biasing, and commitment (Fig. 1A) (Soldatov et al., 2019; Palii et al., 2019; Faure et al., 2020). During the coactivation phase, master TFs from competing lineages are co-expressed in multipotent progenitor cells, a process called multilineage priming (Graf and Enver, 2009; Briggs et al., 2018). During the gradual biasing phase, TF levels change gradually in favor of one of the competing cell fate programs, with alterations in TF stoichiometry tipping the scales towards one fate (Graf, 2019; Gillespie et al., 2020). Finally, the process culminates in mutually exclusive gene expression programs that commit each cell's fate. In doing so, master TFs reinforce their own expression programs, while repressing competing fate programs (Fig. 1A). In other words, cell fate decisions are characterized by different kinds of positive feedback: positive autoregulation (a TF activating itself), double-positive feedback (two TFs of the same fate program activating each other), and double-negative feedback (two TFs of competing fate programs repressing each other; also termed cross-antagonism) (Alon, 2007; Crews and Pearson, 2009; Graf and Enver, 2009; Zhou and Huang, 2011). Master TFs for which positive feedback has been shown to be crucial for fate determination (and whose HI leads to DDs) include *NKX2-5* and *TBX1* during heart development (Molkentin et al., 2000; Hu et al., 2004), *POU3F2*, *RORB*, *SATB2*, and *TBR1* during brain development (Srinivasan et al., 2012; Oishi et al., 2016), *FLI1*, *GATA2*, *RUNX1*, and *TCF4* during hematopoiesis (Pimanda et al., 2007a, b; Grajkowska et al., 2017), and *HNF1A* and *HNF4A* during pancreas development (Odom et al., 2004). A more comprehensive overview can be found in Tables S1 and S3.

Cell fate is mainly controlled by the assembly of master TFs and cofactors at cell-type-specific enhancers, many of which are so-called super-enhancers (SEs) (see Glossary, Box 1) (Spitz and Furlong, 2012; Hnisz et al., 2013; Whyte et al., 2013; Heinz et al., 2015; Reiter et al., 2017). Importantly, SE-associated genes include those encoding the master TFs themselves, thus establishing the autoregulatory positive feedback described above. Compared to normal enhancers, SEs are characterized by a higher number of TF binding sites, which allows for a high density of transcriptional regulators and for extensive cooperative binding of master TFs to DNA (Fig. 1B). Cooperativity results in a sigmoidal (switch-like) transcriptional response (Veitia, 2003), which is particularly suitable for binary decisions such as cell fate choices, but at the same time makes the process extremely sensitive to changes in TF dosage. Cooperativity can be mediated through direct TF–TF interactions, including classic TF dimerization, or through various indirect mechanisms, for example via cofactors (Spitz and Furlong, 2012; Morgunova and Taipale, 2017; Reiter et al., 2017). Many TFs (in their activation domains) and cofactors contain extensive intrinsically disordered regions (IDRs) (see Glossary, Box 1) (Liu et al., 2006; Dyson and Wright, 2016), which provide the basis for cooperativity (Fig. 1B). Based on the large number of cooperative interactions among TFs and cofactors at SEs, Hnisz et al. (2017) proposed a phase separation (see Glossary, Box 1) model for transcriptional control, which is in line with recent findings (Box 3). Master TFs for which cooperative binding has been shown to be crucial for cell fate determination (and whose HI leads to DDs) include *GATA4* and *NKX2-5* during heart development (Durocher et al., 1997), *PAX6* and *SOX2* during eye development (Kamachi et al., 2001), *PAX3* and *SOX10* during neural crest development (Bondurand et al., 2000), *SOX9* during chondrogenesis (Bernard et al., 2003), and *FLI1* and *GATA2* during hematopoiesis (Eisbacher et al., 2003; Shi et al., 2014). A more comprehensive overview can be found in Tables S1 and S3.

**Box 3. Intrinsic disorder, phase separation, and transcriptional condensates**

**Fig. 2.**
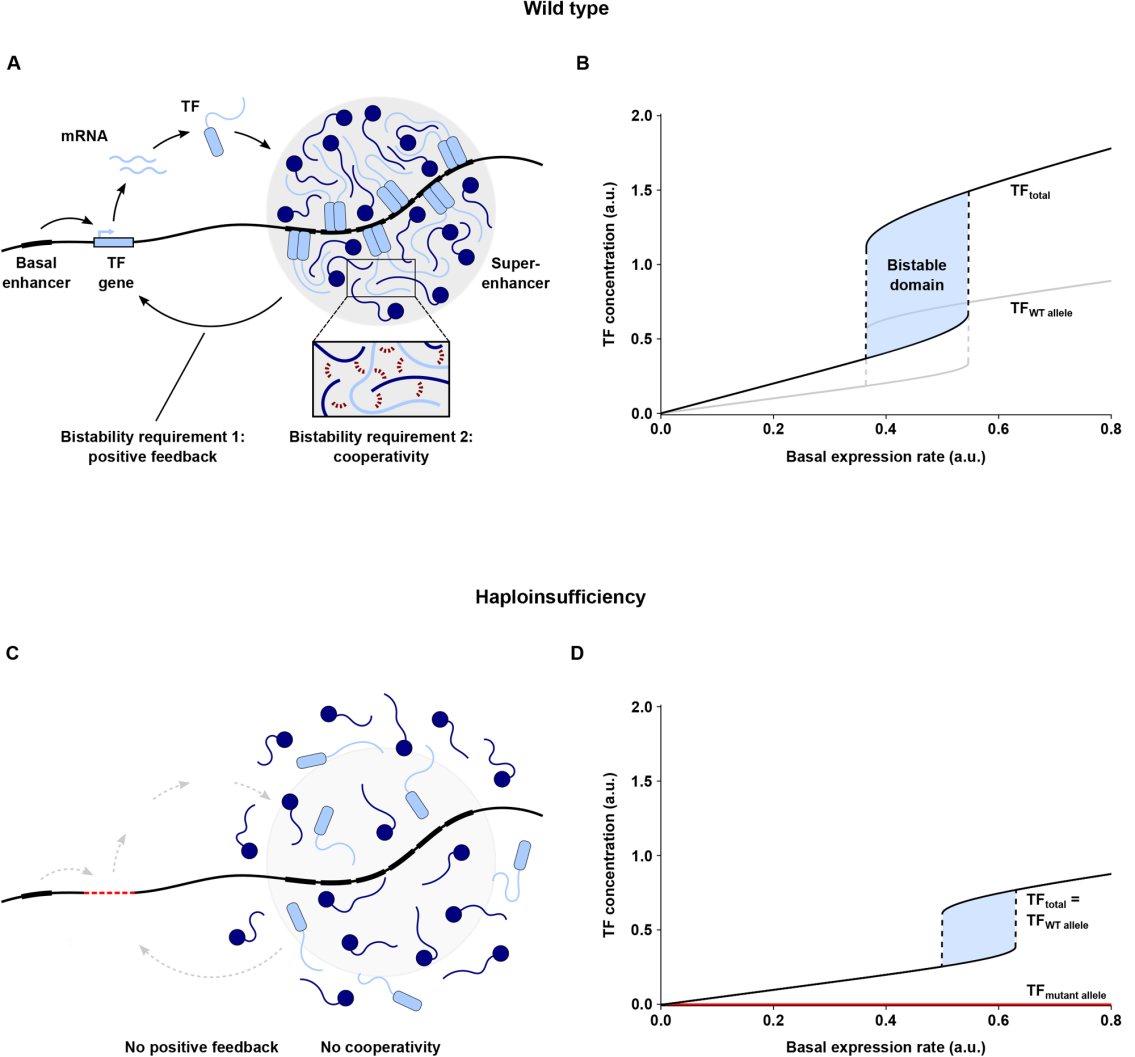
**A model of transcriptional regulation of cell fate and its misregulation due to HI.** (A) Master TFs maintain their own expression through autoregulatory positive feedback by cooperatively binding to their own super-enhancers. Cooperative binding occurs through multivalent interactions between the IDRs of TFs and cofactors (inset) and is presumably associated with the formation of transcriptional condensates (grey circle). (B) When plotting the total TF concentration against the basal expression rate (which stems from a basal enhancer), biallelic (WT) expression generates a bistable domain, where two mutually exclusive cell fates are established by low versus high TF concentration. (C) HI (here caused by TF gene deletion) disrupts positive feedback and cooperativity, as well as condensate formation. (D) HI shifts and diminishes the bistable domain, thus interfering with cell fate determination.

While protein function has long been thought to critically depend on its three-dimensional (3D) structure, it is now clear that there are many proteins for which lack of 3D structure is required for function, so-called intrinsically disordered proteins (IDPs) (see Glossary, Box 1) (Oldfield and Dunker, 2014; Wright and Dyson, 2015). The low complexity sequences characteristic of IDPs provide the basis for multivalent intermolecular interactions. Multivalency – the presence of multiple binding sites on a molecule – enables IDPs to interact with multiple other proteins. Due to dynamic multivalent interactions, IDPs naturally form assemblies, which inherently reduces their solubility. Such assemblies therefore tend to separate into two liquid phases, one being enriched for IDPs and the other one depleted. The process of liquid–liquid phase separation, driven by weak dynamic multivalent interactions between IDP regions, is a general mechanism to form membraneless compartments, so-called biomolecular condensates (see Glossary, Box 1) (Sabari et al., 2020). Phase separation could also explain the formation and function of transcriptional condensates – dynamic multi-molecular assemblies of TFs and cofactors at SEs (Hnisz et al., 2017). Indeed, recent findings corroborate that transcriptional condensates are based on fuzzy and weak cooperative interactions between the IDRs of TFs and cofactors (Fig. 2A) (Boija et al., 2018; Cho et al., 2018; Chong et al., 2018; Sabari et al., 2018; Shrinivas et al., 2019; Sabari, 2020; Ma et al., 2021), and suggest that mutations in the IDRs of transcriptional regulators can lead to aberrant phase separation and impaired formation of transcriptional condensates, thus resulting in DDs (Basu et al., 2020; Fasciani et al., 2020; Tsang et al., 2020).

Taken together, the co-occurrence of positive feedback and cooperativity is a key regulatory motif in the control of cell fate and cell identity (see also Arendt et al., 2016). For convenience, I will term this GRN motif ‘positive feedback with cooperativity’ (PFC) (see Glossary, Box 1). But what exactly does the PFC motif have to do with HI of transcriptional regulators?

### The hypothesis: HI results from disruption of the PFC motif

Putting together the information outlined above, it is possible to sketch a simple model of the transcriptional regulation of cell fate determination (Fig. 2A), which helps to explain the distinct dosage sensitivity (i.e. HI) of transcriptional regulators. Strikingly, this explanation can be derived directly from the features of the PFC motif. It is the high level of cooperativity found at SEs, resulting from the large number of TF binding sites and the high density of transcriptional regulators, that makes the positive feedback loops between master TFs and their SEs particularly sensitive to changes in TF/cofactor concentration (Lovén et al., 2013; Whyte et al., 2013; Hnisz et al., 2017). The dosage sensitivity becomes obvious, for example, during the gradual biasing phase of cell fate determination, when changes in TF stoichiometry will shift cells towards one fate (Palii et al., 2019; Graf, 2019). It also becomes obvious if initial TF levels are below a certain threshold concentration: in this case, the feedback loop will not be triggered. In fact, it is the concept of bistability that explains the occurrence of a threshold in the first place, which corresponds to the unstable steady state separating the two stable steady states.

Taken together, key cell identity genes exhibit a pronounced sensitivity to reduced dosage of the proteins that regulate their expression. In other words, dosage sensitivity of master TFs and cofactors is an inherent feature of cell fate decisions. For the vast majority of human TF/cofactor genes, this dosage sensitivity manifests as HI (for interesting exceptions, see Conclusions and outstanding questions, below). For those genes, heterozygous loss of function is sufficient to disrupt the PFC, leading to cell fate errors and, ultimately, DDs (Fig. 2C). Therefore, I propose the hypothesis that DDs caused by TF/cofactor HI result from disrupted bistability (i.e. disruption of positive feedback or cooperativity) in the PFC motif of the associated cell fate decision (PFC hypothesis, for short). Such disruption may arise from any mechanism producing loss of function (Box 2). In the following two subsections, I will present evidence in support of the PFC hypothesis.

### Empirical evidence

As outlined above, master TFs whose HI leads to DDs are generally involved in positive feedback (Tables S1 and S3). Crucially, for many of these TFs, disruption of positive feedback has been identified as the cause of human disease, including *LMX1B* in nail–patella syndrome (Haro et al., 2021), *PAX6* in aniridia (Bhatia et al., 2013), *SOX10* in Waardenburg syndrome (Lecerf et al., 2014), *SOX9* in sex reversal (Croft et al., 2018), *FOXG1* in FOXG1 syndrome (Ye et al., 2022), *FOXP2* in speech–language disorder (Becker et al., 2018), *NFIA* in a brain malformation syndrome (Trevino et al., 2021), *GATA2* in immunodeficiency (Johnson et al., 2012), and *HNF1A* and *HNF4A* in MODY (Hansen et al., 2002). If disruption of positive feedback is due to loss-of-function mutations in CREs (regulatory loss of function; Box 2), this frequently results in the isolated, rather than the syndromic, form of the disorder. For example, *LMX1B* regulatory loss of function results in limb anomalies rather than full nail–patella syndrome (Haro et al., 2021), *SOX10* regulatory loss of function results in isolated Hirschsprung's disease rather than Waardenburg syndrome (Lecerf et al., 2014), and *FOXG1* regulatory loss of function results in isolated strabismus rather than FOXG1 syndrome (Ye et al., 2022). The reason is that most CREs are tissue-specific and will, when mutated, only affect that tissue.

As with positive feedback, the literature review revealed abundant evidence that master TFs whose HI leads to DDs are involved in cooperative binding (Tables S1 and S3). For many of these TFs, disruption of cooperativity has been identified as the cause of disease, including *GATA4* and *TBX5* in CHDs (Hiroi et al., 2001; Ang et al., 2016), *SOX9* in campomelic dysplasia (Sock et al., 2003), *TWIST1* in Saethre–Chotzen syndrome (Firulli et al., 2005), *SIX1* in branchio-oto-renal syndrome (Ruf et al., 2004), *NR5A1* in sex reversal (Tremblay and Viger, 2003), *TBR1* in ASD (Deriziotis et al., 2014), *BACH2* in immunodeficiency (Afzali et al., 2017), *HNF1A* and *HNF4A* in MODY (Hua et al., 2000; Singh et al., 2019), and *SIM1* in obesity (Sullivan et al., 2014).

### Theoretical evidence

Simple modeling corroborates that TF HI can lead to cell fate errors via disruption of the PFC motif. In many models of cell fate determination, two mutually exclusive cell fates are established by low versus high concentration of a master TF, illustrated by a bistable domain when plotting TF concentration against a parameter, such as the TF basal expression rate (Box 4). In the wild type, expression from both TF alleles leads to a broad bistable domain (Fig. 2B). HI, by contrast, disrupts the PFC for one allele (Fig. 2C; HI illustrated here by gene deletion). However, this also affects expression from the other allele, because TF action is independent of allelic origin; for example, reduced TF concentration (or an inactive TF protein) will affect both enhancers and thus impair expression from both alleles. As a consequence, HI considerably shifts and diminishes the bistable domain of the whole system (Fig. 2D), thus narrowing or eliminating the scope for regular cell fate determination. Modelling other loss-of-function mechanisms (such as reduced gene expression or protein activity; Box 2) leads to similar results (see Supplementary Materials).

**Box 4. A simple model of TF HI**

Here I present a simple model of gene regulation to address TF HI, including both positive feedback and cooperativity, and hence able to generate bistability. The model describes a TF that cooperatively binds to its own enhancer to increase its expression. To address HI explicitly, I consider a single TF locus with two alleles: allele 1 (WT) is controlled by enhancer E_1_ and produces TF variant A_1_, and allele 2 (mutant) is controlled by enhancer E_2_ and produces TF variant A_2_. Note that, under HI, the mutant TF variant A_2_ will often be non-existent, due to gene deletion, enhancer deletion, or NMD. I adopt a fractional occupancy approach in which TF variants compete for occupancy at enhancers (e.g. Porter et al., 2017). The fractional occupancy Θ of a TF describes the proportion of time an enhancer is occupied by a TF molecule; hence, Θ determines the gene expression level from that enhancer. The fractional occupancy depends on [A], the concentration of free TF molecules, and on the binding strength between TF and enhancer, here given as the association constant *K*. The fractional occupancy Θ_A1→E1_ of TF variant A_1_ on enhancer E_1_ in the presence of TF variant A_2_ is given by:
(1)

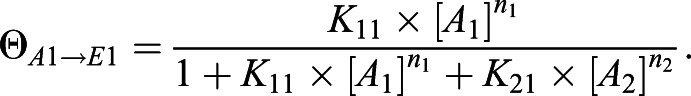
Here, *K*_11_ and *K*_21_ are the association constants between TF variants A_1_ and A_2_ and enhancer E_1_, respectively, and *n* is the Hill coefficient, denoting the degree of cooperativity. Fractional occupancies of the other three interactions are calculated analogously (see Supplementary Material).

The total fractional occupancy at enhancer E_1_ is:
(2)

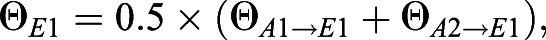
and that at enhancer E_2_ is:
(3)

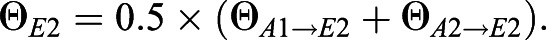
I use ordinary differential equations to describe the change of mRNA and TF concentration over time (for example, Hsu et al., 2016). The mRNA synthesis and decay at allele *i*={1, 2} is given by:
(4)

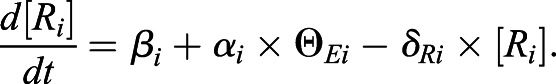
Here, [R] denotes the mRNA concentration, *β* the basal expression rate, *α* the maximum expression rate, and *δ*_R_ the mRNA degradation rate.

The TF synthesis and decay at allele *i*={1, 2} is given by:
(5)

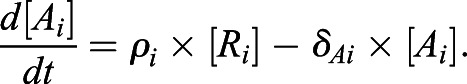
Here, *ρ* denotes the translation rate, and *δ*_A_ the TF degradation rate.

The parameter values used to obtain the results shown in Fig. 2 are given in the Supplementary Material.

In sum, there is strong empirical and theoretical evidence supporting the hypothesis that DDs caused by HI of transcriptional regulators result from disrupted bistability (i.e. disrupted positive feedback or cooperativity) in the underlying GRN. In the following section, I discuss several issues related to DDs for which the PFC hypothesis provides a particularly promising explanatory approach.

## Insights and implications

### Incomplete penetrance and variable expressivity

A notorious finding in medical genetics is that not all carriers of a disease-causing mutation actually develop the disease, and those that express the disease phenotype often do so in different degrees. These phenomena are known as incomplete penetrance and variable expressivity, respectively (Zlotogora, 2003; Cooper et al., 2013), and they are pervasive among DDs (Flannick et al., 2013; Moreno-De-Luca et al., 2013; Kirov et al., 2014; Stefansson et al., 2014; Ropers and Wienker, 2015; Chen et al., 2016; Wright et al., 2019; Kaplanis et al., 2020). A striking example of incomplete penetrance comes from an individual that is healthy despite carrying a disease-causing mutation in *SOX9*, which usually causes campomelic dysplasia and leads to early childhood death (Chen et al., 2016). Both incomplete penetrance and variable expressivity have intrigued clinicians for decades, and their underlying causes are still not well understood. While it seems likely that both genetic and environmental factors contribute (Zlotogora, 2003; Cooper et al., 2013), I shall focus here on the genetic aspects. In particular, I discuss how the gene regulatory PFC motif might increase our understanding of incomplete penetrance and variable expressivity of DDs. Given our comprehensive understanding of differentiation processes in *Caenorhabditis elegans*, I draw on examples from this model organism, but the gene regulatory logic that these examples reveal should apply to humans, as well.

**Fig. 3.**
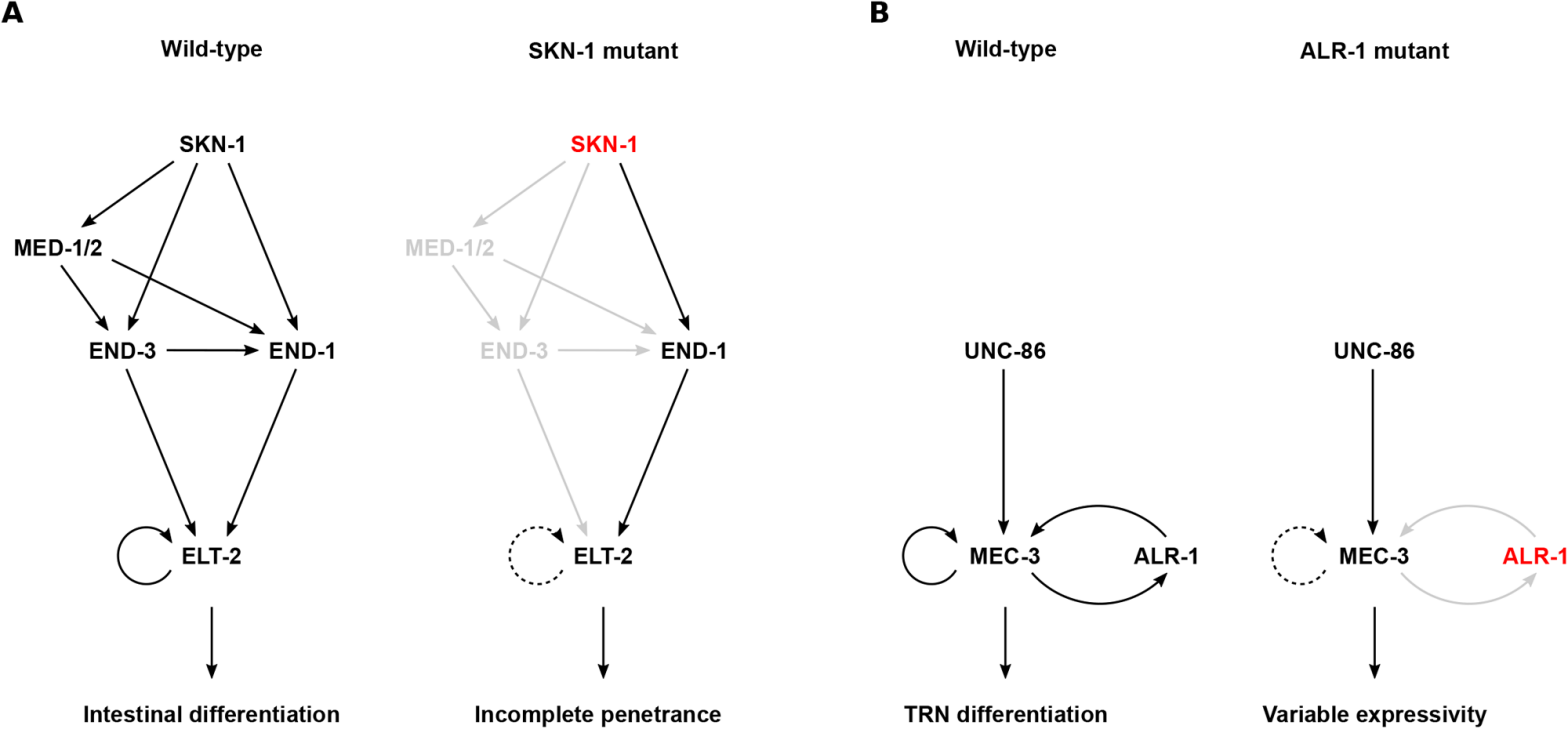
**Incomplete penetrance and variable expressivity during *C. elegans* cell fate determination.** (A) Gene regulatory network (GRN) underlying *C. elegans* intestinal development. In the WT, *end-1* is activated redundantly by SKN-1, MED-1/2 and END-3. This redundancy ensures that *end-1* expression is sufficiently high to trigger *elt-2* auto-activation, and thereby to induce intestinal development. *skn-1* mutations remove this redundancy, resulting in highly variable *end-1* expression, which frequently fails to trigger *elt-2* auto-activation. Adapted from Raj et al. (2010). (B) GRN underlying *C. elegans* touch receptor neuron (TRN) differentiation. In the WT, UNC-86 induces *mec-3* expression, and MEC-3 maintains its own expression in an autoregulatory loop. An additional positive feedback loop involving ALR-1 ensures TRN differentiation by reducing variability of *mec-3* expression. *alr-1* mutations remove the parallel feedback loop, thereby increasing variability in *mec-3* expression. Adapted from Topalidou et al. (2011).

Goldschmidt (1938) explained incomplete penetrance by assuming some threshold effect combined with stochastic fluctuation in gene expression (see also Dietrich, 2003). His prescient view has been corroborated only recently. Not only is the stochastic nature of gene expression now common knowledge (Raj and van Oudenaarden, 2008), Goldschmidt's postulated threshold has been described in some mechanistic detail. Raj et al. (2010) studied incompletely penetrant mutations in the TF gene *skn-1*, which is on top of the GRN controlling intestinal cell fate specification in *C. elegans* (Fig. 3A). The authors showed that *skn-1* mutations bring fluctuations in gene expression within the GRN close to a critical threshold (while wild-type fluctuations are far away from the threshold, because the GRN is buffered due to genes with redundant functions). Strikingly, the threshold was shown to be caused by transcriptional auto-activation of a master TF of intestinal development, ELT-2, which deploys cooperative DNA binding (Neves et al., 2007). Those mutants for which gene expression happens to be too low to trigger the positive feedback loop will display a mutant phenotype (failed intestinal development), while the others will not. Given that the threshold behavior of incomplete penetrance can be easily explained by positive feedback with cooperativity (PFC) – the classic motif of cell fate determination – I suggest that this motif represents a common mechanism underlying the incomplete penetrance distinctive of many DDs.

The combined effect of PFC and stochastic gene expression might also help in explaining variable expressivity. Topalidou et al. (2011) studied mutations in the GRN controlling touch receptor neuron (TRN) differentiation in *C. elegans* (Fig. 3B). TRN fate determination crucially depends on auto-activation of the master TF MEC-3, which binds cooperatively to its own enhancer (Xue et al., 1993). Mutations in *alr-1*, another TF gene in the TRN GRN, result in variable touch receptor function (i.e. variable expressivity), which reflects increased variability in *mec-3* expression. The authors showed that, due to stochastic fluctuations, MEC-3 auto-activation is not sufficient for consistent TRN fate determination. Rather, ALR-1 provides a second positive feedback loop parallel to the autoregulatory MEC-3 feedback loop. This second feedback loop restricts variability in *mec-3* expression and thus ensures TRN fate specification. Such parallel arrangements of positive feedback loops might be a general feature to limit cell-to-cell variability, and hence variable expressivity, during cell fate determination (Ahrends et al., 2014; Dey and Barik, 2017).

Despite cell-to-cell variability due to stochastic gene expression, development is remarkably robust. There is good evidence that GRNs are constructed in a way that minimizes variability and hence increases robustness (MacNeil and Walhout, 2011). As seen above, genetic redundancy and parallel feedback loops within GRNs are examples of such buffering mechanisms. In addition, recent findings suggest that the process of phase separation (Box 3) also contributes to reducing gene expression noise (Klosin et al., 2020). Mutations that interfere with any of these buffering mechanisms (genetic redundancy, parallel feedback, condensate formation) can expose otherwise buffered variability, leading to phenomena such as incomplete penetrance and variable expressivity.

### DDs and the continuum between Mendelian and complex disease

This article focuses on DDs that are caused by rare large-effect loss-of-function variants in specific genes. Hence, many of them would generally be considered as Mendelian (monogenic), rather than complex diseases, which are influenced by multiple genetic and environmental factors. However, there is good reason to regard Mendelian and complex diseases as the two extremes of a continuum, rather than distinct categories (Lupski et al., 2011; Jordan and Do, 2018; Claussnitzer et al., 2020). In fact, complex diseases (and NDDs in particular) are best viewed as umbrella terms for collections of genetically heterogenous conditions, where many different genes are involved across the population, but individual cases are mostly caused by rare, large-effect variants in only one or a few of those genes, modified by the polygenic background of common variants of very small effect (Mitchell, 2015). From this perspective, many complex diseases fall well within the scope of this article. Within the proposed framework, genes affected by large-effect variants would be those directly involved in a PFC motif, while additional genes connected to the PFC motif would contribute indirectly, and to a lesser extent, to disease risk. This view roughly corresponds to the distinction between ‘core genes’ and ‘peripheral genes’ in the omnigenic model of complex traits (Boyle et al., 2017). In the following paragraph, I discuss the important role that rare large-effect variants in transcriptional regulator genes play in the etiology of autism and other NDDs, which shows the utility of the outlined framework for the understanding of such complex disorders.

### The importance of rare large-effect variants in transcriptional regulator genes for the etiology of autism and other NDDs

The umbrella term autism spectrum disorder (ASD) describes a group of highly heritable and heterogeneous NDDs affecting about 1% of individuals (Lord et al., 2020). With hundreds of genes associated with it, ASD is widely regarded as a classic example of a complex disease. In the last ten years or so, tremendous progress has been made regarding the genetic underpinnings of ASD. Most of the proteins encoded by ASD risk genes are involved in either synaptic structure and function or transcriptional regulation and chromatin remodelling; examples of the latter include *ARID1B*, *BCL11A*, *CHD8*, *FOXP1*, *KMT2C*, *MBD5*, *TBR1*, *TCF4*, and *TCF20* (Sestan and State, 2018; Iakoucheva et al., 2019; Sullivan et al., 2019; Lord et al., 2020; Satterstrom et al., 2020). Strikingly, there is insufficient evidence that any of these genes is ‘autism-specific’; in fact, there is no gene known that, when mutated, confers risk only for ASD and not for other NDDs (Myers et al., 2020; see also below). Furthermore, risk genes are predominantly affected by rare inherited heterozygous loss-of-function mutations, strongly indicating HI as the mechanism of pathogenesis (Krumm et al., 2015; Ji et al., 2016; Sestan and State, 2018; Sullivan et al., 2019; Lord et al., 2020). In addition, noncoding regulatory mutations also contribute to ASD risk, and those noncoding regions affect the same haploinsufficient genes previously found to be implicated in ASD (Zhou et al., 2019). Overall, the emerging picture is that HI of transcriptional regulators is one of the main pathogenic mechanisms underlying ASD.

Recent years have also seen substantial progress in unravelling the genetics of other NDDs, including ID, schizophrenia, and bipolar disorder (BD). As with ASD, many risk variants are rare inherited loss-of-function mutations affecting haploinsufficient genes involved in transcriptional regulation and chromatin remodelling, but also haploinsufficient noncoding regulatory regions (Short et al., 2018; Turner and Eichler, 2019; Moyses-Oliveira et al., 2020; Parenti et al., 2020). Interestingly, it has become clear that pathogenic variants in the same TF/cofactor genes are involved in various NDDs, suggesting a shared genetic risk across disorders (Moreno-De-Luca et al., 2013; Owen and O'Donovan, 2017; The Brainstorm Consortium, 2018; Wray et al., 2018; Grove et al., 2019; Satterstrom et al., 2019; Stahl et al., 2019; Myers et al., 2020; Lee et al., 2021). For example, HI of *MBD5* is associated with ASD, BD, and ID (Hodge et al., 2014), HI of *MEF2C* with ASD, epilepsy, ID, and speech delay (Le Meur et al., 2010; Novara et al., 2010), and HI of *SETD1A* with ASD, ID, and schizophrenia (Singh et al., 2016; Kummeling et al., 2021). Taken together, the importance of rare, large-effect variants in specific TF/cofactor genes for the etiology of ASD and other NDDs not only underlines the continuum between Mendelian and complex disease, but also suggests that the proposed framework represents a promising explanatory approach.

### Testing the PFC hypothesis

The PFC hypothesis states that DDs caused by HI of cell-fate-determining master regulators result from disruption of bistability in the GRN of that fate decision. Since bistability is a mathematical concept and not observable experimentally, it is difficult to test this hypothesis directly. However, it is possible to test its corollary, that is, whether DDs result from disruption of positive feedback or cooperativity in the GRN. The repeated identification of such disruptions as the underlying cause of disease in affected individuals provides already strong evidence in support of this prediction (see above), and future research will surely provide further support. Furthermore, one can test experimentally whether, for a TF known to cause a DD via HI, inhibition of positive feedback or cooperativity leads to the same developmental defects as those caused by HI of that TF. If this is the case, it would lend support to the hypothesis. Recently, human brain organoids have emerged as an exciting experimental model system to study the origins of NDDs (Baldassari et al., 2020; Chan et al., 2020). Brain organoids could also be used to experimentally test the corollary of the PFC hypothesis. For example, downregulation of *NR2F1*, a TF gene whose HI causes the NDD Bosch–Boonstra–Schaaf optic atrophy syndrome, impairs neural differentiation in human brain organoids (Bertacchi et al., 2020). Future experiments could use genetic modification tools such as CRISPR/Cas9 to inhibit NR2F1 positive feedback or cooperativity in brain organoids. If this disruption of the PFC motif elicits similar neural differentiation defects as those seen after *NR2F1* downregulation, this would support (albeit not proof) the PFC hypothesis. Similar experiments could be done with other TFs that are known to cause DDs through HI. In the long run, findings from such experiments could also contribute to novel therapeutic approaches that modulate gene regulation to treat HI-associated DDs (Matharu and Ahituv, 2020).

## Conclusions and outstanding questions

DDs are a universal human concern (Wallingford, 2019). There is now broad evidence that many DDs, however diverse in their clinical manifestations, share a common cause, that is, they result from HI of TFs and chromatin modifiers. Why transcriptional regulators exhibit such a pronounced dosage sensitivity is still unclear. Building upon existing theories to explain HI, I have outlined an explanatory framework, at the heart of which is a systems biology perspective on cell fate determination. I have argued that dosage sensitivity of transcriptional regulators is an inherent feature of cell fate decisions, and I have proposed the hypothesis that DDs result from disrupted bistability (i.e. disrupted positive feedback or cooperativity) in the GRN of the underlying fate decision. While such a perspective provides novel insights, there remain important outstanding questions and problems for future research. Here I will briefly discuss some of them.

In order to better understand how HI of transcriptional regulators leads to disease, it will be crucial to quantitatively measure the effects of heterozygous loss-of-function mutations on protein levels. Many studies have shown such mutations to reduce TF protein levels using semi-quantitative methods, such as western blotting (Yousfi et al., 2001; Jay et al., 2005; Liu et al., 2018; Kathiriya et al., 2021). However, for a detailed understanding of HI-associated disease, it will be necessary to measure the absolute number of TFs in single cells, and methodologies to do so are already within reach (Auer et al., 2020).

What is the exact role of phase separation in transcriptional regulation of cell fate? As described above (Box 3), there is a growing body of literature linking transcriptional activation at SEs to the formation of transcriptional condensates, driven by liquid–liquid phase separation (reviewed by Sabari, 2020). However, this idea has not gone unchallenged (McSwiggen et al., 2019; Mir et al., 2019). For example, it is still unclear whether condensate formation *per se* enhances transcription (Trojanowski et al., 2021 preprint). In fact, alternative mechanisms other than phase separation exist to drive transcription, including classic cooperative binding of TFs to DNA and the assembly of well-defined multi-subunit protein complexes. It will be crucial to determine the contribution of each of these processes to the transcriptional control of cell fate.

Why does HI of chromatin modifiers predominantly affect brain development (Table S2), although these regulators are generally expressed in most cell types? The development and functioning of the brain, with its myriad cell types, might put exceptional demands on gene expression, which must allow for both stable maintenance and flexible fine-tuning of neuronal circuitries. Creating this balance between flexibility and stability might make chromatin regulation particularly important in brain development (Ronan et al., 2013). Neuron-specific alternative splicing of chromatin regulator genes might be an important mechanism to achieve the unique gene expression patterns the brain demands, and might at least partially explain why chromatin regulator HI so often leads to NDDs (Porter et al., 2018).

Do haploinsufficient TF/cofactor genes also show sensitivity to increased dosage (triplosensitivity)? The PFC hypothesis makes no predictions about this issue. Whereas in yeast, haploinsufficient genes seem to be also triplosensitive (Morrill and Amon, 2019), a recent dosage-sensitivity analysis of the human genome revealed several features that distinguish haploinsufficient from triplosensitive genes, such as insulation from other genes and higher local cis-regulatory complexity (Collins et al., 2021). The fact that these features are characteristic of master TFs suggests that such TFs are not triplosensitive. Nevertheless, it might be worthwhile to extend the presented model to include triplosensitivity.

Why do some master TFs not show HI? A few DDs are caused by homozygous (or compound heterozygous) loss of function of master TF genes. In other words, these genes do not show HI, and the associated diseases are recessive, in contrast to those discussed in this article. Examples include *HOXA1* and *HOXB1* (Tischfield et al., 2005; Vogel et al., 2016), *NKX3-2* (Hellemans et al., 2009), *PAX1* and *PAX7* (Feichtinger et al., 2019; Pohl et al., 2013), *PRDM12* (Chen et al., 2015), *PTF1A* (Sellick et al., 2004), as well as *TBX15* and *TBX19* (Couture et al., 2012; Lausch et al., 2008). It seems likely that these genes show a dosage sensitivity that is less pronounced than HI, as a loss of function stronger than 50% is necessary to elicit a disease phenotype. The degree of dosage sensitivity strongly depends on the degree of cooperativity, which in turn depends on the number of TF binding sites in a gene's CREs (Fig. 1B). Perhaps, the CREs of the aforementioned genes have less TF binding sites and therefore show a lower dosage sensitivity.

Why are there differences in gene dosage sensitivity between species, especially between humans and mice? Such differences are frequently observed in TF genes underlying human DDs. Generally, heterozygous loss-of-function mutations in these genes show a disease phenotype in humans, but not in mice; in other words, only humans are haploinsufficient for these genes. As with the previous question, it seems probable that the difference in dosage sensitivity stems from differences in the cis-regulatory architecture. In particular, the discrepancy might be related to the evolutionary divergence of CREs between the two species, and to the consequences of this for TF binding (Odom et al., 2007; Han et al., 2018). It is tempting to speculate that a higher number of TF binding sites in humans (as compared to mice) led to higher dosage sensitivity.

How does dosage sensitivity evolve in the first place? In general, the evolution of gene regulation is driven not only by changes in CREs (e.g., TF binding sites, see above), but also in the TF proteins themselves (Lynch and Wagner, 2008; Cheatle Jarvela and Hinman, 2015). Indeed, as dosage sensitivity is mainly determined by the degree of TF cooperativity, investigating the evolution of protein cooperativity might be helpful in addressing this question. Existing studies indicate that cooperativity can evolve through just a few mutations, by exploiting pre-existing structural features of the protein (Nnamani et al., 2016; Pillai et al., 2020). Does this mean that dosage sensitivity can also evolve in a few steps, by modification of pre-existing features of TF structure?

## Supplementary Material

Supplementary information
