# Supplementary material for: Developmental disorders caused by haploinsufficiency of transcriptional regulators: a perspective based on cell fate determination
Source: Biol Open. 2022 Jan 28;11(1):bio058896. doi: 10.1242/bio.058896 (PMC8801891; doi:10.1242/bio.058896)
Supplement: Supplementary information [file biolopen-11-058896-s1.pdf]

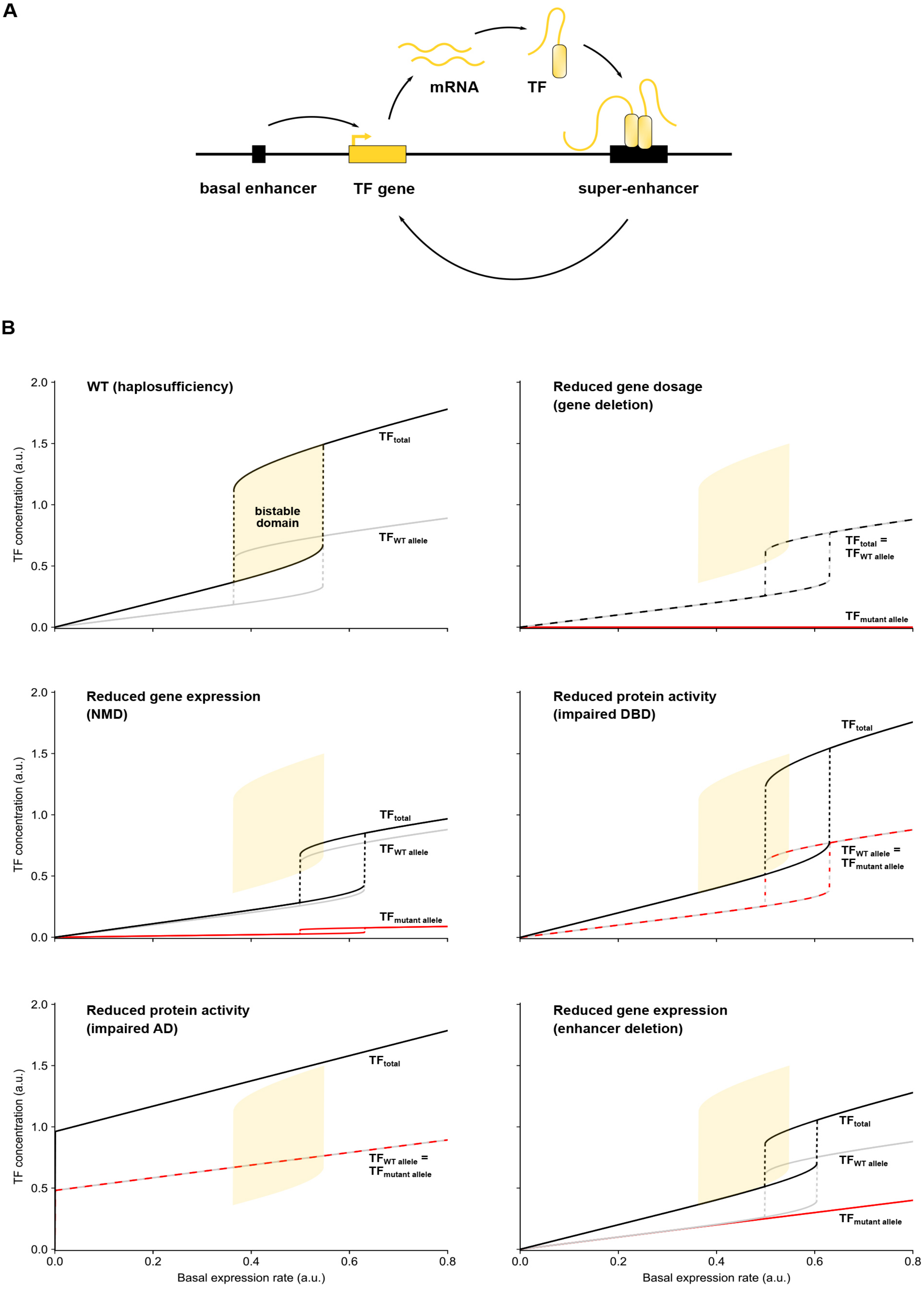

Biology Open • Supplementary information

Fig. S1. Modeling results for the WT and different HI scenarios. (A) Simplified schematic of the positive feedback loop with cooperativity (PFC), which can generate bistability. (B) Plots show the WT and different HI scenarios. Upper panel: WT and HI due to reduced gene dosage (gene deletion); these two plots are also shown in Fig. 2 in the main text. Middle panel: HI due to reduced gene expression (nonsense-mediated mRNA decay [NMD]) and to reduced protein activity (impaired DNA-binding domain [DBD]). Lower panel: HI due to reduced protein activity (impaired activation domain [AD]) and to reduced gene expression (enhancer deletion). Total TF concentration is shown in black, TF concentration from the WT allele in grey, and TF concentration from the mutant allele in red. For comparison, the bistable domain (in light yellow) is shown in all plots.

The fractional occupancies (see main text) are calculated as follows:

$$\Theta_{A1\rightarrow E1}=\frac{K_{11} * [A_1]^{n_1}}{1+K_{11} * [A_1]^{n_1}+K_{21} * [A_2]^{n_2}}$$

$$\Theta_{A2\rightarrow E1}=\frac{K_{21} * [A_2]^{n_2}}{1+K_{11} * [A_1]^{n_1}+K_{21} * [A_2]^{n_2}}$$

$$\Theta_{A1\rightarrow E2}=\frac{K_{12} * [A_1]^{n_1}}{1+K_{12} * [A_1]^{n_1}+K_{22} * [A_2]^{n_2}}$$

$$\Theta_{A2\rightarrow E2}=\frac{K_{22} * [A_2]^{n_2}}{1+K_{12} * [A_1]^{n_1}+K_{22} * [A_2]^{n_2}}$$

In order to obtain the results shown in Figure S1 and in Figure 2 in the main text, TF concentration was solved numerically at steady state and plotted against the basal expression rate β. The following parameter values were used:

WT (haplosufficiency):
$\alpha_1 = \alpha_2 = 1$, $K_{11} = K_{21} = K_{12} = K_{22} = 50$, $n_1 = n_2 = 6$, $\rho_1 = \rho_2 = 1$, $\delta_{R1} = \delta_{R2} = \delta_{A1} = \delta_{A2} = 1$.

HI due to reduced gene dosage (gene deletion):
same as WT, but $\beta_1 = \alpha_1 = 0$, $\beta_2 = 0.5 * \beta_{total}$.

HI due to reduced gene expression (NMD):
same as WT, but $\delta_{R1} = 10$.

HI due to reduced protein activity (impaired DBD):
same as WT, but $K_{11} = K_{12} = 1$.

HI due to reduced protein activity (impaired AD):
same as WT, but $n_1 = 1$.

HI due to reduced gene expression (enhancer deletion):
same as WT, but $\alpha_1 = 0$.

Biology Open • Supplementary information

Table S1.

Click here to download Table S1

Table S2.

Click here to download Table S2

Table S3.

Click here to download Table S3

Biology Open • Supplementary information
